# Characterisation of plasmid-mediated *rmtB-1* in *Enterobacteriaceae* clinical isolates from São Paulo, Brazil

**DOI:** 10.1590/0074-02760180392

**Published:** 2018-12-10

**Authors:** Dandara Cassu-Corsi, Willames MBS Martins, Adriana G Nicoletti, Luiz GP Almeida, Ana TR Vasconcelos, Ana C Gales

**Affiliations:** 1 Universidade Federal de São Paulo, Escola Paulista de Medicina, Departamento de Medicina Interna, Divisão de Doenças Infecciosas, Laboratório Alerta, São Paulo, SP, Brasil; 2 Laboratório Nacional de Computação Científica, Petrópolis, RJ, Brasil

**Keywords:** aminoglycosides resistance, 16S rRNA methyltransferases, K. pneumoniae harboring rmtB-1, P. mirabilis harboring rmtB-1, *Enterobacteriacea rmtB-1* coproducing *bla*_KPC-2_, outbreak

## Abstract

OBJECTIVES The emergence of 16S rRNA methyltranferases (16 RMTAses) has jeopardised the clinical use of aminoglycosides. RmtB is one of the most frequently reported in Gram-negatives worldwide. In this study, we aimed to estimate the frequency of 16S RMTAses encoding genes in *Enterobacteriaceae* isolated in a three-month period from a tertiary Brazilian hospital. METHODS All Gram-negatives classified as resistant to amikacin, gentamicin, and tobramycin by agar screening were selected for analysis. The presence of 16SRMTases encoding genes was verified by polymerase chain reaction (PCR). Antimicrobial susceptible profile was determined by broth microdilution. The genetic relationship among these isolates was accessed by pulsed field gel electrophoresis (PFGE) and multilocus sequence typing (MLST). Selected RmtB-producing isolates were characterised by whole genome sequencing (WGS) analysis. RESULTS Twenty-two of 1,052 (2.1%) *Enterobacteriaceae* were detected as producers of RmtB-1 [*Klebsiella pneumoniae* (n = 21) and *Proteus mirabilis* (n = 1)]. _*blaKPC-2*_ was identified among 20 RmtB-1-producing *K. pneumoniae* isolates that exhibited an identical PFGE and MLST (ST258) patterns. Two *K. pneumoniae* isolates, the A64216 (not harboring *bla*_KPC-2_), A64477 (harboring *bla*_KPC-2_) and one *P. mirabilis* isolate (A64421) were selected for WGS. *rmtB-1* and *bla*_KPC-2_ genes were carried by distinct plasmids. While a plasmid belonging to the IncFIIk group harbored *rmtB-1* in *K. pneumoniae*, this gene was carried by a non-typable plasmid in *P. mirabilis*. In the three analysed plasmids, *rmtB-1* was inserted on a transposon, downstream a Tn*2*. CONCLUSION Our findings suggested that the *rmtB-1* was harbored by plasmids distinct from those previously reported in Bolivia and China. It suggests that multiple mobilization events might have occurred in South America.

*Enterobacteriaceae* are the most frequent pathogens associated with both community- and hospital-acquired infections. In the last years, the emergence of carbapenemase production in *Enterobacteriaceae* has jeopardised the clinical use of carbapenems.^1^ In this way, polymyxins and aminoglycosides have become therapeutic options for treatment of serious infections caused by carbapenem resistant *Enterobacteriaceae* (CRE). With the emergence of polymyxin resistance especially among KPC-2-producing isolates, the aminoglycosides have reached even a more important role in the treatment of CRE infections.^2^ Resistance to aminoglycosides is often due to the production of aminoglycoside modifying enzymes (AMEs), which usually confer resistance to specific aminoglycoside molecules but not all aminoglycosides.^3^ In contrast, the production of 16S RMTases will confer high-level of resistance to 4,6 - dissubstituted 2-deoxystreptamines aminoglycosides including plazomicin, a new antimicrobial not approved for clinical use yet.^3^ ^,^ ^4^ To date, ten 16S RMTAses (ArmA, RmtA to RmtH, and NpmA) have been detected in Gram-negative isolates.^4^ ArmA is the most frequently reported 16S RMTAses worldwide followed by RmtB.^4^ ^,^ ^5^ The mobilisation and transfer of 16S RMTAses encoding genes by mobile genetic elements have contributed for their rapid global dissemination.^4^ ^,^ ^5^

In Brazil, the production of 16S RMTases has been mainly encountered in SPM-1 producing *Pseudomonas aeruginosa* (RmtD). *rmtD-2* has been also detected in *Klebsiella pneumoniae*.^4^ In addition, *K. pneumoniae* (*bla*_KPC-2_ and *rmtG*; *bla*_NDM-1_ and *rmtC*) and *Enterobacter cloacae* (*bla*_NDM-1_and *armA*) co-harboring carbapenemase and 16S RMTases encoding genes were also reported.^4^ ^,^ ^5^ ^,^ ^6^. The presence of *rmtB-1* in *Escherichia coli* and *Proteus mirabilis* was also reported in the years 2005 and 2006, respectively.^7^

F33:A-:B-plasmids carrying *bla*_CTX-M-65_, *fosA3* and *rmtB* are widespread in *Escherichia coli* isolates of animal origin from China. pHN7A8 is a representative of this plasmid group and was isolated from a Chinese dog. Curiously, the plasmids p397Kp and p477Kp, which were isolated from multi-drug resistant *K. pneumoniae* ST726 collected from Bolivian patients, showed to be highly related to pHN7A8 suggesting an intercontinental spread of this mobile genetic elements.^8^ In this study, we aimed to estimate the frequency of 16S RMTAses encoding genes in *Enterobacteriaceae* isolated in a three-month period from a tertiary Brazilian hospital. In addition, the plasmids harboring *rmtB-1* of selected isolates were fully sequenced.

## MATERIALS AND METHODS

*Bacterial strains* - All *Enterobacteriaceae* isolated by the microbiology laboratory of a tertiary teaching hospital located in the city of São Paulo, Southeast of Brazil, were selected for this study between October and December of 2014. In order to select the resistant isolates to aminoglycosides, agar screening test was performed by testing agar plates supplemented with gentamicin (4 mg/L), tobramycin (4 mg/L), and amikacin (16 mg/L). The isolates classified as resistant to all three aminoglycosides were selected for further testing. The isolates were identified by MALDI-TOF MS (Bruker Daltonics, Germany) using BioTyper 3.1.^9^

*Investigation of 16S RMTAses β-lactamases encoding genes and sequencing* - Detection of 16S RMTAses encoding genes was performed by two distinct multiplex polymerase chain reaction (PCR) for isolates screened as not susceptible to aminoglycosides by agar screening. PCR multiplex 1: *npmA*, *armA*, *rmtB*, *rmtC* and *rmtD*. PCR multiplex 2*: rmtE, rmtF, rmtG* e *rmtH.* A single PCR was tested for *rmtA* detection [Supplementary data (Table II)]. Investigation of *bla*_TEM_, *bla*_SHV_, *bla*_CTX-M_, *bla*_GES_, *bla*_KPC_ was performed as previously reported ^10^ ^,^ ^11^. Amplicons were purified and sequenced using the Applied Biosystems 3500 genetic analyser (Applied Biosystems, PerkinElmer, USA). The obtained sequences were compared with those available at GenBank (http://www.ncbi.nlm.nih.gov/Blast.cgi).

*Antimicrobial susceptibility testing* - The minimal inhibitory concentrations (MICs) for kanamycin, amikacin, gentamicin, tobramycin, aztreonam, cefepime, ceftazidime, ceftriaxone, ertapenem, imipenem, meropenem, piperacillin/tazobactam, ciprofloxacin, polymyxin B, and tigecycline were determined by broth microdilution for the isolates producers of 16S RMTases. The results were interpreted according to EUCAST clinical breakpoints, except for kanamycin results, which were interpreted according to CLSI breakpoints.^12^ ^,^ ^13^

*Molecular typing* - All *K. pneumoniae* isolates carrying *rmtB-1* were typed by pulsed field gel electrophoresis (PFGE) using Spe-I as restriction enzyme,^14^ and multilocus sequence typing (MLST).^15^

*Plasmid profile and transference of 16S rRNA methyltransferases and bla*_*KPC*_ - Total plasmid DNA extraction of the *K. pneumoniae* and *P. mirabilis* isolates harboring *rmtB-1* was performed using the Kieser technique^16^ and the Qiaprep spin miniprep (Qiagen, Hilden Germany). Four isolates (A64192, A64216, A64477 and A64421) carrying *rmtB-1* and distinct beta-lactamases encoding genes were further selected for both conjugation and transformation. Conjugation experiments were performed using the clinical isolates as donor and *E. coli* J53 as the receptor strains. Cells were grown on MacConkey agar plates supplemented with azide or nalidixic acid (150 mg/L) plus amikacin (8 mg/L) or ampicillin (50 mg/L) for selection of transconjugant cells carrying *rmtB-1* or β-lactamase encoding genes, respectively. Additionally, the DNA plasmids obtained of the extraction by QIAprep spin miniprep were transferred by electroporation and transformation into *E. coli* DH5α. The transformant cells were selected according to the colony growth on Luria Bertani agar (LB agar) plates supplemented with amikacin (8 mg/L), imipenem (1 mg/L) or ampicillin (50 mg/L) for selection of colonies carrying 16S RMTases or β-lactamase encoding genes.

*Southern Blot Hybridisation* - Total DNA and plasmidial DNA of clinical isolates were used to perform Southern Blot Hybridisation using dioxigenin (DIG) DNA labeling and detection kit (Roche Diagnostics GmbH, Germany).

*Whole genome sequencing, assembly, annotation, and analysis* - Two RmtB-1-producing *K. pneumoniae* isolates, the A64216 (not harboring *bla*_KPC-2_), A64477 (harboring *bla*_KPC-2_) and one RmtB-1-producing *P. mirabilis* isolate (A64421) were selected for whole genome sequencing. To obtain a better coverage of the plasmidial against chromosomal DNA, the extraction was performed by commercial kit PerfectPrep Spin Mini Kit (5 prime, Gaithersburg, Maryland), and shipped to the National Laboratory for Scientific Computing (LNCC - Petrópolis, RJ, Brazil), where the experiments were carried out. The library was constructed by Illumina TruSeq DNA PCR-free with a fragment ~550pb and sequenced by Illumina MiniSeq 2x300 pb paired-end. The sequences were assembled using Newbler version 3.0 and Ray version 2.1. The contigs were aligned with the blast against the NT database for separation of plasmid DNA from chromosomal DNA. The coverage of the chromosomes ranged from 14x to 40x, while plasmid coverage ranged from 700x to 20,000x. The system for automated bacterial (genome) integrated annotation (SABIA) pipeline^17^ was used for gene prediction and automatic annotation followed by manual validation of each predicted CDS by Uniprot (http://www.uniprot.org/), BLAST (http://blast.ncbi.nlm.nih.gov). Insertion sequences were searched by using ISfinder (https://www-is.biotoul.fr) after automatic annotation fallowed by manual validation. The comparison of the similarity genetic and Inc group analysis were performed by Multiple Genome Alignment (MAUVE) and PlasmidFinder (hhtps://cge,cbs.dtu.dk//services/PlasmidFinder/), respectively. Additionally, the investigation of the resistance genes harbored by other plasmids was evaluated by ResFinder program (https://cge.cbs.dtu.dk/services/ResFinder/). The size confirmation of *rmtB-1* were confirmed by S1-PFGE,^18^ followed by radioactive hybridisation.

*Nucleotide sequence accession numbers* - The nucleotide sequences of the plasmids were deposited in the GenBank under accession numbers, pKP64477a (MF150084), pKP64477b (MF150122), pKP64477c (MF150121), pKP64477d (MF150120), pKP64477e (MF150119), pKP64216a (MF135602), pKP64216b (MF150123), pKP64216c (MF150124), pPM64421a (MF150118) and pPM64421b (MF150117).

## RESULTS

Total of 1,052 *Enterobacteriaceae* were recovered during the period of study. Twenty-two were classified as resistant to aminoglycosides by agar-screening, and all of them were detected as possessing *rmtB-1*. These isolates were identified as *K. pneumoniae* (n = 21) and *P. mirabilis* (n = 1) as listed in Table. All *Enterobacteriaceae* isolates showed high levels of resistance to amikacin (MIC, > 256 mg/L), gentamicin (MIC, > 256 mg/L), kanamycin (MIC, > 256 mg/L), and tobramycin (MIC, > 128mg/L). High resistance rates to ciprofloxacin (MIC, > 16 mg/L) and broad-spectrum cephalosporins (MICs, 64-> 128 mg/L) were also observed. Twenty of 21 RmtB*-*1-producing *K. pneumoniae* isolates also harbored *bla*_KPC-2_ and *bla*_CTX-M-15._ Curiously, four *K. pneumoniae* isolates also carried a second cefotaximase encoding gene, *bla*_CTX-M-14_ (Table). The presence of *bla*_TEM-1b_, *bla*_CTX-M-14_ and *bla*_CTX-M-15_ genes was also detected in the single RmtB-1-producing *P. mirabilis.*

Susceptibility to carbapenems was observed only in *K. pneumoniae* (A64216) and *P. mirabilis* (A64421) isolates, which did not harbor *bla*_KPC-2_. All *K. pneumoniae* isolates were resistant to polymyxin B, except for the A64022 and A64962 isolates. In contrast, tigecycline was the antimicrobial that exhibited the highest in vitro potency (MICs, 0.03-0.125 mg/L), and susceptibility rate with only two isolates being categorized as resistant (MICs, 2 mg/L) to this compound as shown in Table. All *K. pneumoniae* showed an identical PFGE pattern and belonged to the ST258. The plasmid profile obtained by Kieser methodology followed by Southern Blot/Hybridisation assay showed that *rmtB-1* was located in a plasmid of similar size in all *K. pneumoniae* isolates. Transfer of *rmtB-1* by conjugation was successfully performed only for the *P. mirabilis* strain (A64421). Transformation experiments testing *K. pneumoniae* as a donor of *rmtB-1* were unsuccessful despite many attempts.

**Fig. 1:**
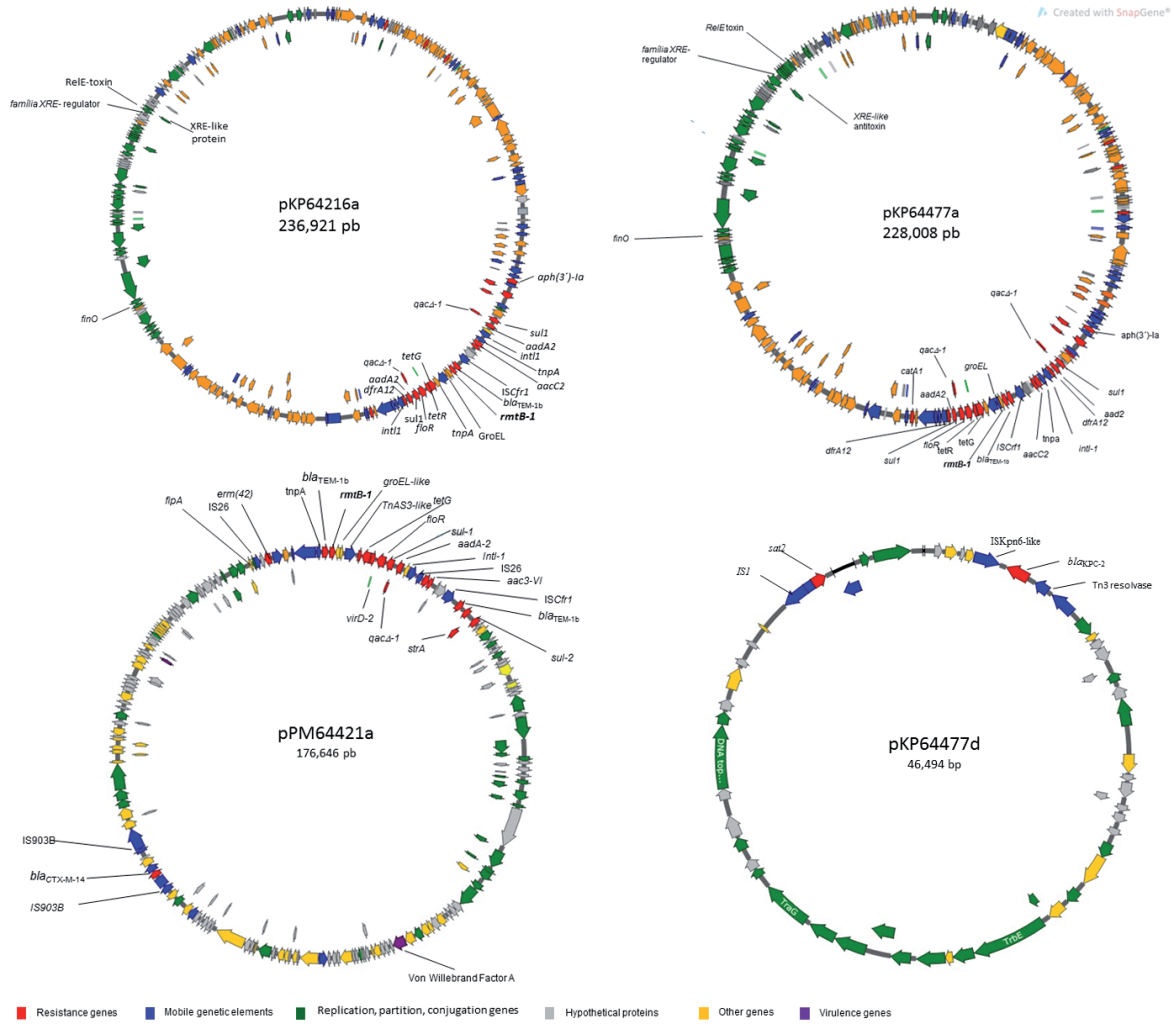
circular map depicting the genetic structures of plasmids harboring *rmtB-1* (pKP64216a, pKP64477a, and pPM64421a) and *bla*_*KPC-2*_ (pKP64477d).

**Fig. 2:**
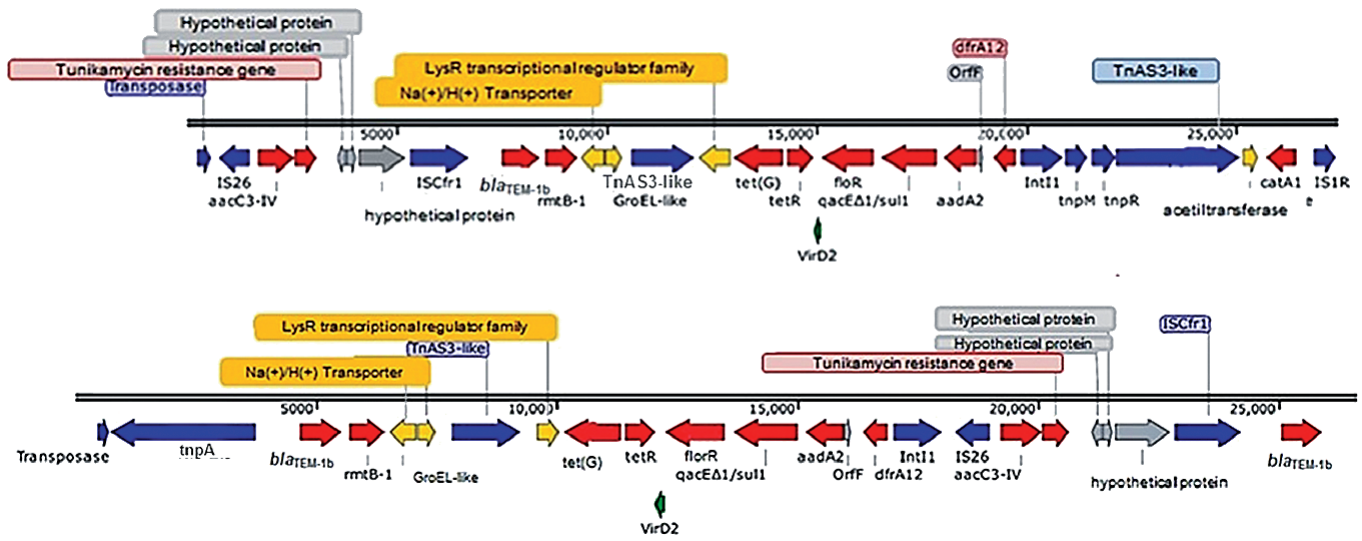
comparison of the *rmtB-1* genetic context displayed by the two *Klebsiella pneumoniae* (A64477 and A64216) and *Proteus mirabilis* (A64421) isolates.

According to plasmid assembly of *K. pneumoniae* and *P. mirabilis* isolates (Fig. 1), the A64216, A64477, and A64421 isolates harbored three (236.9 kb,154.4 kb, and 9,6 kb), five (228 kb, 205 kb, 205 kb,154.,5 kb, 46.4 kb and 9.2 kb) and two plasmids (176.3 kb, and 36 kb), respectively [Supplementary data (Table I)]. General features of the pKP64216a, pKP64477a pKP64477d and pPM64421a were exhibited in Supplementary data (Table I) In *K. pneumoniae* A64477, *bla*_KPC-2_ was located on a distinct plasmid of 46 Kb, which was named pKP6447d [Supplementary data (Table III), Fig. 1]. The pKP64216a and pKP64477a, which harbor *rmtB-1*, showed high nucleotide similarity and basically differ by an 8.913 bp insertion, which was inserted along ~200000 to 209000 regions of the pKP64216a. *rmtB-1* was located upstream of Tn*2* transposon (*tnpA*-*tnpR*-*bla*_TEM-1b_) (Fig. 2). Next to this region, a class 1 integron, In*27*, possessing in its variable region, *aadA2, orf*F and *dfrA*12, respectively, was detected in both pKP64216a and pKP64477a. The sequence comparation of pKP64477a, pKP64216a and pPM64421 were exhibited in Supplementary data (Figure).

*In-silico* analysis showed that both pKP64477a and pKP64216a belonged to IncFIIk group of incompatibility group, while pPM64421 was a non-typable plasmid according to PlasmidFinder analysis.^19^

pKP64477a and pKP26216a showed a distinct backbone, when compared with pPM464421a, except for the region in which *rmtB-1* was inserted. In this region, an insertion of IS*Cfr1* upstream *rmtB-1* region in pKP64416a and pKP64477a was observed. While, a *tnpA* gene was detected in the pPM64421a. Various aminoglycoside-modifying enzymes encoding genes were detected in these plasmids, such as *aadA2*, *aph(3’)*, *aac(3’)-IId*, *strA* and *strB*. In addition, other resistance genes such as *sul1*, *sul2*, *drfA12*, *tetG*, *erm(42)*, and *catA,* which confer resistance to sulphonamides, trimethroprim, tetracycline, macrolides, and phenicol, respectively, were also detected [Supplementary data (Tables I, III)]. Only two virulence encoding genes were observed on pPM64421a that carried a Fe^3+^ siderophore ABC transporter permease and a Von Willenbrand factor A.

The incompatibility group of the pKP64477d, which harbors *bla*_KPC-2_ could not be fully typed. *In-silico* analysis suggested that this plasmid could belonged to InX3 (99.7%) or IncU (99.5%) group. pKP64477d carried only another resistance gene, *sat2*, which conferred resistance to streptomycin (Fig. 1). The *bla*_KPC-2_ was inserted into a transposon, flanked by an IS*Kpn26* and a Tn*3* resolvase.

## DISCUSSION

Aminoglycosides have been an important therapeutic option for treatment of serious Gram-negative multi-drug resistant infections.^4^ However, the emergence and spread of 16S RMTAses capable of conferring high level resistance to aminoglycosides has jeopardised the clinical use of this important class of antimicrobials. In Brazil, SPM-1-producing *P. aeruginosa* ST277 clone harboring *rmtD-1* has been frequently detected justifying the high level of aminoglycoside resistance displayed by this MDR clone.^20^ 16S RMTAse encoding genes were sporadically detected in Latin America before the year 2007, and basically restricted to the transfer of *rmtD* to non-*P. aeruginosa* species.^4^ However, contemporary studies have reported the emergence of RmtG and RmtB in *K. pneumoniae* isolated from Brazil and Bolivia, respectively.^4^ ^,^ ^20^ ArmA and RmtC were also recently identified in NDM-1-producing *Enterobacter cloacae*,^5^ and - *K. pneumoniae* isolates.^6^ Despite the low prevalence of 16S RMTases detected in our hospital, we observed that the increase in frequency of the 16S RMTases could be mainly attributed to the intra-hospital spread of a single RmtB-1-producing *K. pneumoniae* clone.

RmtB-1-producing Gram-negative isolates usually harbor distinct enzymatic mechanisms of resistance, such as β-lactamases, AMES and PMRQ.^4^ ^,^ ^5^ The isolates characterised in this study also accumulated distinct mechanisms of resistance, like production of β-lactamases (KPC-2, CTX-M-14, CTX-M-15, SHV-11 and TEM-1) and diverse AMEs (AADA2, APH(3’)-Ia, AAC(3)-IId, StrA, StrB). All RmtB-1-producing *K. pneumoniae* isolates belonged to ST258 (clonal complex CC258), which has been often detected in KPC-2-producing *K. pneumoniae* isolated in Brazil.^21^

To date, pKP644216a and pKP64477a were distinct from other plasmids harboring *rmtB-1*. Both plasmids showed 99% of similarity with a partial nucleotide sequence (~70 Kb) of pKPN-IT (accession number JN233704).^22^ This ~70 Kb region did not harbor *rmtB-1*, suggesting that recombination events had occurred allowing the acquisition of *rmtB-1* by both Brazilian plasmids. Additionally, pKP64216a and pKP64477a belonged to IncFIIk, an uncommon group associated with *rmtB-1* dissemination.^23^ In contrast, the nucleotide sequences of pPM64421a showed 99% identity with a partial nucleotide sequence (~90 Kb) of a *Vibrio cholareae* plasmid pVC1307 (accession number KJ817377), which did not harbor *rmtB-1* either*,* suggesting that *rmtB-1* was mobilised and integrated to this genetic backbone. Although, the plasmids carrying *rmtB-1* have complete conjugation machinery in their genetic backbone,^20^ unsuccessful transfer of *rmtB* has been reported. *finO* was identified in the plasmids pKP644477a and pKP64216a, which is an inhibitor of *traJ*, and could have circumvented the genetic material transfer from *K. pneumoniae* to the recipient strain.^24^

**TABLE Microbiologic:** features of *RmtB-1*-producing *Klebsiella pneumoniae* and *Proteus mirabilis* isolated from distinct patients

Isolate number	MALDI-TOF ID	Medical units	Month/Year of isolation	Body source	MIC (mg/L)															β-lactamase content
					AMK	GEN	TOB	KAN	FEP	CAZ	CRO	IPM	MEM	ETP	CIP	TZP	ATM	PMB	TGC	
A63760	*K. pneumoniae*	Adults ICU	Oct./2014	Blood	>256	>256	>128	>256	64	128	>256	128	>128	>128	>16	>256/4	>128	16	0.125	KPC-2; TEM-1b; CTX-M-15; SHV-11
A63901	*K. pneumoniae*	Adults ICU	Oct./ 2014	Tracheal aspirate	>256	>256	>128	>256	64	128	>256	128	>128	>128	>16	>256/4	>128	16	0.125	KPC-2; TEM-1b; CTX-M-14; CTX-M15; SHV-11
A63934	*K. pneumoniae*	Adults ICU	Oct./2014	Urine	>256	>256	>128	>256	64	128	>256	2	32	32	16	>256/4	>128	32	0.03	KPC-2; TEM-1b; CTX-M-14; CTX-M15; SHV-11
A64022	*K. pneumoniae*	Adults ICU	Oct./2014	Urine	>256	>256	>128	>256	64	64	>256	64	>128	>128	>16	>256/4	>128	0.125	0.125	KPC-2; TEM-1b; CTX-M-15; SHV-11
A64102	*K. pneumoniae*	Adults ICU	Dec./2014	Urine	>256	>256	>128	>256	64	128	>256	128	64	>128	>16	>256/4	>128	2	0.03	KPC-2; TEM-1b; CTX-M-15; SHV-11
A64192	*K. pneumoniae*	Adults ICU	Oct./2014	Tracheal aspirate	>256	>256	>128	>256	64	64	>256	32	64	>128	>16	>256/4	>128	64	0.125	KPC-2; TEM-1b; CTX-M-14; CTX-M15; SHV-11
A64216	*K. pneumoniae*	Adults ICU	Oct./2014	Urine	>256	>256	>128	>256	64	64	>256	0.125	1	8	>16	>256/4	>128	64	0.125	TEM-1b; CTX-M-15; SHV-11
A64315	*K. pneumoniae*	Adults ICU	Nov./2014	Blood	>256	>256	>128	>256	>128	128	>256	>128	64	>128	>16	>256/4	>128	32	1	KPC-2; TEM-1b; CTX-M-14; CTX-M15; SHV-11
A64343	*K. pneumoniae*	Adults ICU	Nov./2014	Blood	>256	>256	>128	>256	>128	128	>256	128	64	>128	>16	>256/4	>128	8	1	KPC-2; TEM-1b; CTX-M-15; SHV-11
A64345	*K. pneumoniae*	Adults ICU	Nov./2014	Blood	>256	>256	>128	>256	128	128	>256	>128	>128	>128	>16	>256/4	>128	32	2	KPC-2; TEM-1b; CTX-M-15; SHV-11
A64403	*K. pneumoniae*	Adults ICU	Nov./2014	Tracheal aspirate	>256	>256	>128	>256	64	64	>256	4	32	64	16	>256/4	>128	32	0.03	KPC-2; TEM-1b; CTX-M-14; CTX-M15; SHV-11
A64406	*K. pneumoniae*	Adults ICU	Nov./2014	Surgical wound	>256	>256	>128	>256	64	64	>256	32	64	128	16	>256/4	>128	8	0.03	KPC-2; TEM-1b; CTX-M-15; SHV-11
A64477	*K. pneumoniae*	Adults ICU	Nov./2014	Urine	>256	>256	>128	>256	64	>128	>256	16	64	128	>16	>256/4	>128	32	0.03	KPC-2; TEM-1b; CTX-M-15; SHV-11
A64520	*K. pneumoniae*	Adults ICU	Nov./2014	Secretion	>256	>256	>128	>256	>128	64	>256	128	128	>128	>16	>256/4	>128	64	1	KPC-2; TEM-1b; CTX-M-15; SHV-11
A64522	*K. pneumoniae*	Adults ICU	Nov./2014	Other	>256	>256	>128	>256	>128	128	>256	>128	>128	>128	>16	>256/4	>128	64	2	KPC-2; TEM-1b; CTX-M-15; SHV-11
A64611	*K. pneumoniae*	Adults ICU	Dec.2014	Urine	>256	>256	>128	>256	>128	128	>256	>128	128	>128	>16	>256/4	>128	64	0.5	KPC-2; TEM-1b; CTX-M-15; SHV-11
A64774	*K. pneumoniae*	Adults ICU	Dec./2014	Urine	>256	>256	>128	>256	>128	128	>256	128	128	>128	>16	>256/4	>256	64	0.5	KPC-2; TEM-1b; CTX-M-15; SHV-11
A64922	*K. pneumoniae*	Adults ICU	Dec./2014	Urine	>256	>256	>128	>256	64	64	>256	32	32	128	>16	>256/4	>256	4	0.03	KPC-2; TEM-1b; CTX-M-14; CTX-M15; SHV-11
A64962	*K. pneumoniae*	Adults ICU	Dec./2014	Urine	>256	>128	>128	>256	>128	64	>256	32	>128	>128	>64	>256/4	>128	64	1	KPC-2; TEM-1b; CTX-M-15; SHV-11
A64971	*K. pneumoniae*	Adults ICU	Dec./2014	Urethral secretion	>256	>256	>128	>256	>128	>128	>256	32	64	128	16	>256/4	>256	0.125	0.03	KPC-2; TEM-1b; CTX-M-15; SHV-11
A65030	*K. pneumoniae*	Adults ICU	Dec./2014	Blood	>256	>256	>128	>256	64	64	>256	32	32	64	16	>256/4	>256	2	0.03	KPC-2; TEM-1b; CTX-M-15; SHV-11
A64421	*P. mirabilis*	Adults ICU	Nov./2014	Blood	256	>256	>128	>256	>128	>128	>256	32	0.6	≤0.5	>16	16/4	32	>64	NT	TEM-1b; CTX-M-14; CTX-M-15;

AMK: amikacin; ATM: aztreonam; CAZ: ceftazidime; CIP: ciprofloxacin; CRO: ceftriaxone; ETP: ertapenem; FEP: cefepime; GEN: gentamicin; IPM: imipenem; ICU: intensive care unit; KAN: kanamycin; MALDI-TOF ID: matrix-assisted laser desorption/ionization time-of-flight mass spectrometry; MEM: meropenem; MIC: minimal inhibitory concentration; PMB: polymyxin B; TGC: tigecycline; TOB: tobramycin; TZP: piperacillin/tazobactam.

The genetic contexts of pKP644477a and pKP64216a were similar to previous description,^25^ which showed *bla*_TEM-1_ upstream *rmtB*. Our results showed that ∆*Tn*2 [IS*Cfr1*-∆*tnpR*-*bla*_TEM-1b_] was inserted upstream *rmtB-1* in pKP64216a and pKP64477, while a Tn*2* [*tnpA*-*tnpR*-*bla*_TEM-1b_] was present upstream *rmtB-1* in pPM64421. The region surround ∆Tn*2* in pKP644477a and pKP64216a showed high similarity with other transposons carried by pU302L (Accession number: AY333434) and pCTX-M-3 plasmids [(Accession number: AF550415)].^26^ ^,^ ^27^ In contrast to what was previously reported by Sennati and colleagues,^8^ who suggested an intercontinental dissemination of plasmids harboring *rmtB*, the plasmids harboring *rmtB-1* in Brazil were distinct from those observed in Bolivia and China [(p397Kp and p477Kp); (pHN7A8)],^8^ ^,^ ^28^ respectively. The *rmtB-1* genetic context was also different, since both p397Kp (Accession number: LN897474) and p477Kp (Accession number: LN897475) had an insertion sequence IS*1294* upstream Δ*tnpR*, while both pKP644477a and pKP64216a showed an IS*Cfr1* upstream Δ*tnpR*.^8^

It is curious to note that the gene encoding the VonWillebrand factor A, a virulence factor only observed in *S. aureus* to date, was detected in these large plasmids. VonWillebrand factor A is responsible for protecting the bacteria from the neutrophilic attack.^29^ Toxin-antitoxin system was observed in the pKP64477a and pKP64216a, the RelE-XrE-like. A few studies have evaluated the role of RelE-XrE-like system, which has been associated with cellular stress response being expressed, for example, when nutrients are limited.^30^

In addition to *bla*_KPC-2_, pKP64477d was similar to pKP13d, a previously sequenced plasmid harboring *bla*_KPC-2,_ collected from another Brazilian state (accession number NZ_CP003997). The single difference observed between these two plasmids was the acquisition of 917 bp, which comprised basically the insertion of IS*26*. The acquisition of *rmtB-1* by a KPC-2-producing *K. pneumoniae* ST258 clone could justify the success of *rmtB-1* spread. This illustrates how dynamic is the evolution of antimicrobial resistance warranting the need for continuous surveillance.
