# Supplementary material for: Characterisation of plasmid-mediated rmtB-1 in Enterobacteriaceae clinical isolates from São Paulo, Brazil
Source: Mem Inst Oswaldo Cruz. 2018 Dec 10;113(12):e180392. doi: 10.1590/0074-02760180392 (PMC6287189; doi:10.1590/0074-02760180392)
Supplement: Supplementary file 1 [file 1678-8060-mioc-113-12-e180392-s.pdf]

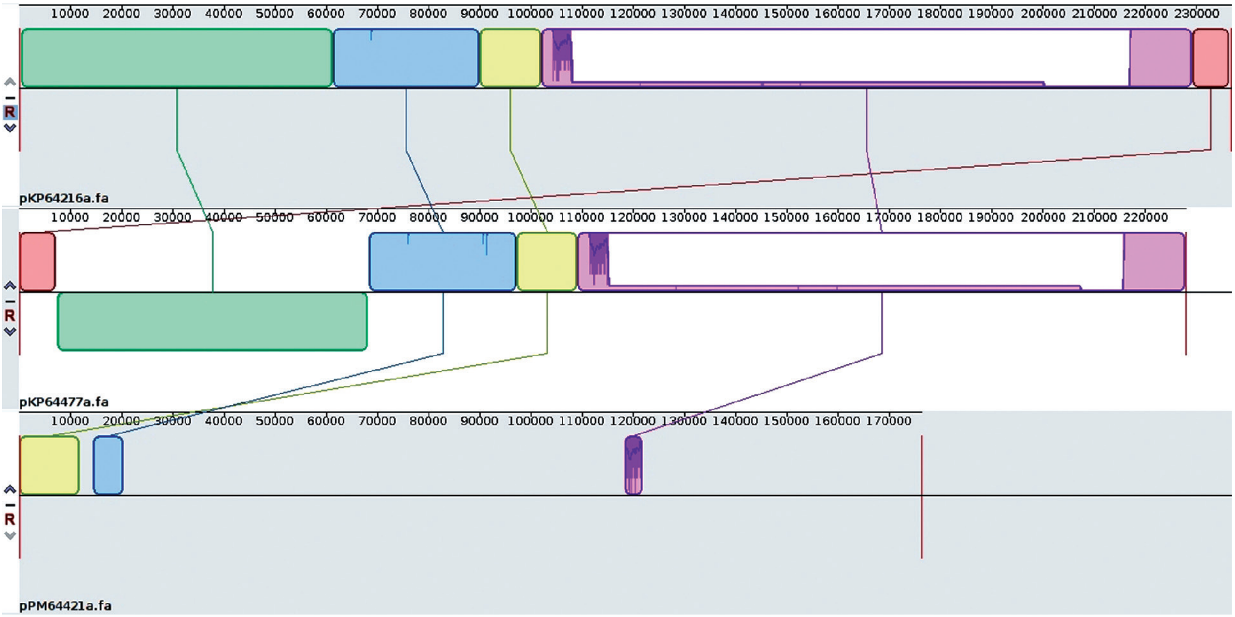

Sequence comparation of pKP64477a, pPM64421a and pKP64216a. Figure generated by MAUVE Genome Alignment Visualisation.

TABLE I

General features of the plasmids harboring *rmtB-1* (pKP64216a, pKP64477a, and pPM64421a) and bla_{KPC-2} (pKP64477b)

	Plasmids			
Features	pKP64216a	pKP64477a	pKP64477d	pPM64421a
Base pairs	236,921 bp	228,008 bp	46,494 bp	176,346 bp
GC content	53,41%	53.38%	46.1%	51.9%
Resistance genes	22	23	2	17
Mobile genetic elements	43	35	5	16
Mobile, conjugation and replication genes	45	42	20	37
Virulence genes	1	1	_	2
Hypothetical proteins	56	50	21	94
Other genes	89	85	10	44
ORFS total	256	236	58	210

CC BY

TABLE II

Sequence of primers tested for 16S RMAtases detection

		Sequence (5'-3')	Size (bp)	Reference
**PCR Multiplex 1-** 16S RMTases	NpmA-F<br>NpmA-R	GGA GGG CTA TCT AAT GTG GT<br>GCC CAA AGA GAA TTA AAC TG	386	(14)
	ArmA-F<br>ArmA-R	ATT CTG CCT ATC CTA ATT GG<br>ACC TAT ACT TTA TCG TCG TC	316	This study<br>(14)
	RmtB-F<br>RmtB-R	GAA TGG GGG CGG CAT AAA TC<br>AAG TTC TGT TCC GAT GGT CTT	643	This study
	RmtC-F<br>RmtC-R	CGA CGT GTA ACT GAG AGG CTT<br>TCG CCT GAC GGA TCG GAT AA	500	This study
	RmtD-F<br>RmtD-R	GAC CGA GCG CGA ATA CAA AC<br>CGG AAA CGA TGC GAC GAC GAT	440	This study<br>(14)
**PCR Multiplex 2-** 16S RMTases	RmtE-F<br>RmtE-R	GGA GGG CTA TCT AAT GTG GT<br>GCC CAA AGA GAA TTA AAC TG	355	This study
	RmtF-F<br>RmtF-G	ATT CTG CCT ATC CTA ATT GG<br>ACC TAT ACT TTA TCG TCG TC	434	This study
	RmtG-F<br>RmtG-R	CTA GCG TCC ATC CTT TCC TC<br>TTG CTT CCA TGC CCT TGC C	535	This study
	RmtH-F<br>RmtH-R	GAA TGG GGG CGG CAT AAA TC<br>AAG TTC TGT TCC GAT GGT CTT	653	This study
**PCR Single-** 16S RMTases	RmtA-F<br>RmtA-R	CTA GCG TCC ATC CTT TCC TC<br>TTG CTT CCA TGC CCT TGC C	762	(14)

The 16S RMTases polymerase chain reaction (PCR) detection with the followed parameters: 94°C - 5 min; 94°C - 20min; 60°C -1 min; 72°C - 75 s; 35 cycles; 72ºC-10 min.

TABLE III

Description of the resistance genes found in all plasmids identified in the A64216, A64477, and A64421 clinical isolates

Bacteria isolate	Plasmid	Size	Coverage	Resistance gene[a]	Antimicrobial resistance class	Contig number	Accesion number
K. pneumoniae A64216[d]	pKP64216a	236.9 kb	700x	*aadA2* (2 copies)	Aminoglycoside	100774 - 101565/80367 - 81158	JQ364967
				aph(3')-Ia	Aminoglycoside	71015 - 71830	V00359
				aac(3)-IId	Aminoglycoside	84433 - 85293	EU022314
				rmtB-1	Aminoglycoside	91295 - 92050	AB103506
				bla_{TEM-1b}	β-lactam	90265 - 91125	JF910132
				mph(A)	Macrolide	72807 - 73712	D16251
				catA1	Phenicol	108481 - 109140	V00622
				sul1 (2 copies)	Sulphonamide	79023 - 79949/ 99741 - 100356	CP002151
				dfrA12 (2 copies)	Trimethoprim	81566 -82063/101973 - 102470	AB571791
				tet(G)	Tetracycline	95755 - 96930	AF133140
	pKP64216b	154.4 kb	780x	*strA*[b]	Aminoglycoside	89217 - 90020	AF321551
				strb[c]	Aminoglycoside	90020 - 90856	M96392
				bla_{TEM-1b}	β-lactam	91271 - 92131	JF910132
				erm(42)	Macrolide	99273 - 100184	AB601890
				sul2	Sulphonamide	88341 - 89156	GQ421466
	pKP64216c	9,6 kb	20000x	-	-	-	-
K. pneumoniae A64477[e]	pKP64477a	228 kb	700x	*aadA2* (2copies)	Aminoglycoside	87504-88295/107911-108702	JQ364967
				aph(3')-Ia	Aminoglycoside	78152-78967	V00359
				aac (3')-IId	Aminoglycoside	91570-92430	EU022314
				rmtB	Aminoglycoside	98432-99187	AB103506
				bla_{TEM-1b}	β-lactam	97402-98262	JF10132
				mph(A)	Macrolide	79944-80549	D16251
				catA1	Phenicol	115622-116281	V00622
				sul1(2 copies)	Sulphonamide	106878-107493/86160-87086	CP002151
				tet(G)	Tetracycline	102892-104019	AF071555
				dfrA12(2 copies)	Trimethoprim	109110-109607/88703-89200	AB571791
	pKP64477b	205 kb	440x	-	-	-	-
	pKP64477c	154,5 kb	900x	*strA*[b]	Aminoglycoside	90276-91079	AF321551
				strB[c]	Aminoglycoside	91079-91915	M96392
				bla_{TEM-1b}	β-lactam	92330-93190	JF910132
				erm(42)	Macrolide	100332-101243	AB601890
				sul2	Sulphonamide	89400-90215	GQ421466
	pKP64477d	46,4 kb	2700	bla_{KPC-2}	β-lactam	3034-3915	AY034847
	pKP64477e	9,2 kb	12000x	-	-	-	-
P. mirabilis A64421[f]	pKP64421a	176.3 kb	2500x	*aadA2*	Aminoglycoside	10702-11493	JQ364967
				rmtB	Aminoglycoside	1223-1978	AB103506
				aac(3)-IId	Aminoglycoside	14768-15628	EU022314
				strB[c]	Aminoglycoside	21875-22711	M96392
				strA[b]	Aminoglycoside	22711-23514	AF321551
				$bla_{CTX-M-14}$	β-lactam	114386-115261	AF252622
				bla_{TEM-1b}(2 copies)	β-lactam	193-1053/0600-21460	JF10132
				erm(42)	Macrolide	168476-169387	AB601890
				sul2	Sulphonamide	23575-24390	GQ421466
				sul1	Sulphonamide	9669-10194	JN581942
				tet(G)	Tetracycline	5683-6858	AF133140
				dfrA12	Trimethoprim	11901-12398	AB71791
	pKP64421b	36 kb	9500x	-	-	-	-

a: the resistance genes identified by Resfinder program; *b*: alternative name, *aph(3'')-Ib*; *c*: alternative name *aph(6)-Id*; *d*: *Klebsiella pneumoniae* A64216 chromosome coverage: 30x; *e*: *K. pneumoniae* A64477 chromosome coverage: 14x; *f*: *Proteus mirabilis* A64421 chromosome coverage: 40x.
